# The impact of COVID-19 on South African public-sector interventional radiology services – A single-centre study

**DOI:** 10.4102/jcmsa.v4i1.233

**Published:** 2026-01-30

**Authors:** Ursula Lesar, Michelle Da Silva, Richard Pitcher

**Affiliations:** 1 Department of Medical Imaging and Clinical Oncology, Faculty of Medicine and Health Sciences, Stellenbosch University, Cape Town, South Africa

**Keywords:** interventional radiology, COVID-19, procedures, pandemic, public hospital

## Abstract

**Background:**

Little is known about the impact of coronavirus disease 2019 (COVID-19) on South African interventional radiology (IR) services. This study aimed to assess the influence of COVID-19 on IR procedures at a tertiary-level public sector South African (SA) hospital.

**Methods:**

A retrospective audit of IR procedures over three 9-week periods in 2020: Period (1) pre-strict lockdown (23 January–25 March), period (2) strict lockdown (26 March–27 May) and period (3) post-strict lockdown (28 May–30 June). Data were captured and stratified by patient demographics, procedure indication, nature (vascular or non-vascular), time (normal or after-hours) and location (IR suite or ward). Calculated incidence rates of categories per period were performed and then compared between the three periods.

**Results:**

There were 288, 218 and 204 procedures performed in periods 1, 2 and 3, respectively, with no significant proportional variation in gender, age, after-hours or ward procedures across the periods. During period 2, the overall (*n* = 218, *p* = 0.002), non-vascular (*n* = 148, *p* = 0.001) and vascular procedures (*n* = 70, *p* = 0.999) decreased by 24.3%, 29.8% and 9.1%, respectively. During period 3, the overall (*n* = 204, *p* = 0.496) and non-vascular (*n* = 122, *p* = 0.590) procedures declined by a further 6.4% and 17.6%, respectively, while vascular procedures (*n* = 82, *p* = 0.410) increased by 17.1%. In period 3, the overall (*n* = 204, *p* = 0.001) and non-vascular procedures (*n* = 122, *p* ≤ 0.001) were 29.1% and 42.2% lower than period 1 levels, whereas vascular procedures (*n* = 82, *p* ≥ 0.999) demonstrated the so-called ‘rebound phenomenon’, exceeding period 1 by 6.5%.

**Conclusion:**

COVID-19 pandemic impacted non-vascular and vascular IR procedures with variable difference between periods of strict lockdown and post-strict lockown. This affords a perspective on the emerging role of IR in health systems across the African continent.

**Contribution:**

This study provides unique insights into the impact of COVID-19 on SA IR services.

## Introduction

Severe acute respiratory syndrome coronavirus 2 (SARS-CoV-2) is the highly contagious and virulent causative organism of coronavirus disease 2019 (COVID-19). Although primarily a respiratory disease, COVID-19 has been implicated in a wide range of systemic inflammatory responses, with the potential for multi-organ failure.^1^ The World Health Organization declared COVID-19 a pandemic on 11 March 2020.^1^ The pandemic saw a surge of global hospital admissions, with the potential to overwhelm even the most well-resourced healthcare systems.^2^ Countries responded by invoking public restrictions to limit the spread of infection.^3^

The first South African (SA) COVID-19 case was reported on 05 March 2020, a state of national disaster was declared on 15 March 2020, and from 26 March 2020, the SA Government invoked the *Disaster Management Act of 2002*, with five lockdown levels.^3,4^

Level 5, the strictest, was implemented from 26 March 2020 to 30 April 2020 (Table 1), during which all citizens, except essential workers, were confined to their homes for the entire 24 h day.^5^ Schools and non-essential businesses were closed, and conferences, concerts, sporting events, religious gatherings, and travel (domestic and international) were suspended. Alcohol sales were prohibited. Mask-wearing and social distancing of at least 1.5 m were compulsory in all public spaces.^6^

**TABLE 1:** South African lockdown restrictions per level.

2020 lockdown periods	Level 5	Level 4	Level 3
Time frame	26 March 2020–30 April 2020	01 May 2020–31 May 2020	01 June 2020–17 August 2020
Curfew hours	Citizens confined to homes 24 h per day, except for essential workers and services	21:00–04:00	22:00–04:00
Work in the agriculture, forestry, mining, financial and professional services	Complete prohibition	No limitation	No limitation
School and university activities	Closed	Closed	Open, with limitations
Air travel	International and domestic banned	International and domestic banned	International banned. Domestic permitted if fulfilling employment responsibilities, moving residence, and for academic purposes, funeral attendance, medical treatment. Banned for leisure purposes.
Interprovincial travel	Banned	Banned except for transportation of goods and exceptional circumstances, such as funerals, with a permit	Partial restrictions, same as domestic air (with an authorised permit)
Public transport	Banned	70% capacity for long-distance travel (> 200 km); 100% for short-distance travel (< 200 km)	Yes, 70% capacity for long-distance travel (> 200 km); 100% for short-distance travel (< 200 km)
Alcohol sales	Banned	Limited periods or days of purchasing	No restriction

*Source*: Mahoney SH, Steyn E, Lategan H. Informing future policy for trauma prevention: The effect of the COVID-19 ‘National state of disaster lockdown’ on the trauma burden of a tertiary trauma centre in the Western Cape of South Africa. Afr J Emerg Med. 2021;11(3):361–365. https://doi.org/10.1016/j.afjem.2021.06.002^8^

McKee J. The effect of COVID-19 on interventional radiology around the globe [homepage on the Internet]. c2022 [updated 25 April 2022; cited 2022 May 30]. Available from: https://www.rsna.org/news/2022/april/COVID-And-Interventional-Radiology^9^

During level 4 (01–31 May 2020) (Table 1), some restrictions were eased, and most non-essential businesses were opened. Citizens could leave their homes, but a 21:00–04:00 curfew was invoked.^7^ Alcohol could be sold during restricted hours.

Level 3 (01 June 2020–17 August 2020) (Table 1) saw further relaxation of restrictions. Most non-essential businesses were fully operational. Schools and universities were opened. Funerals and weddings were permitted but with restricted numbers, and there was free movement within provinces. There was further relaxation of the restrictions pertaining to alcohol sales. Although public transport was in operation, occupancy was limited to 70%.^5^

In summary, Levels 4 and 5, considered ‘strict lockdown’, were characterised by reduced social interactions, decreased road traffic, and restricted alcohol consumption, while Level 3 allowed more freedom of movement with resumption of more normal social interaction.

The strict lockdowns imposed in response to the COVID-19 pandemic impacted the patient profile in health facilities globally. Trauma-related emergencies decreased, elective surgical lists were curtailed, and outpatient clinics were closed to allow deployment of personnel to isolation wards and intensive care units to meet the surge in COVID-related emergencies.^9,10,11,12^ In South Africa, during the strict lockdown, hospital emergency admissions decreased by approximately 50%, largely reflecting the lower trauma load.^10,11,12,13,14^

A 2020 systematic review of the impact of COVID-19 on interventional radiology (IR) workload internationally documented widely discrepant (17% – 80%) decreases in institutions across North America, Europe and China. These marked differences were ascribed to institutional variations in study periods, case definition, health care systems and health policy. Broadly, elective and outpatient procedures were more affected than emergencies,^15,16,17^ and there was a tendency to preserve interventional oncology services.

A further feature of the COVID-19 era has been the slow return of patient numbers to pre-COVID-19 levels. A United States (US) study showed the number of patients accessing hospital care remained low throughout 2020, returning to pre-COVID-19 levels in the second quarter of 2021.^18^ During 2021, SA emergency unit attendance (83%), medical admissions (80%), surgical admissions (87%), oncology programme registrations (91%) and outpatient general practitioner visits (96%) were all proportionally lower when compared to pre-COVID-19 levels.^19^ In South Africa, the number of patients accessing hospital care remained low throughout 2020 and 2021. However, it improved year-on-year in relation to pre-COVID-19 levels (2019).^19^ This trend is demonstrated in Table 2; however, return to baseline has not been defined in the SA setting.

**TABLE 2:** Annual percentage of health service utilisation compared to 2019 (pre-pandemic levels), in a South African private care setting.

Service	2020 (%)	2021(%)
Emergency department	68.1	83.4
Medical hospital admissions	64.1	79.5
Surgical hospital admissions	72.6	87.0
Out-of-hospital GP visits	85.5	95.9
Oncology programme registration	81.8	91.1

*Source*: South African government. Disaster Management Act: Declaration of a national state of disaster: COVID-19 (coronavirus) [homepage on the Internet]. c2020 [updated 2020; cited 2022 Mar 18]. Available from: https://www.gov.za/documents/disaster-management-act-declaration-national-state-disaster-covid-19-coronavirus-16-mar^20^

GP, general practitioner physician.

Tygerberg Hospital (TBH) in Cape Town is South Africa’s second largest hospital, with 1386 beds and a drainage population of approximately 3.6 million people.^21^ Pre-COVID-19, its Level 1 Trauma Unit managed 12 000–15 000 seriously injured persons per year, and its 24-h IR service managed approximately 1500 cases annually.^11^

In South Africa, IR is a relatively new, but rapidly expanding field which utilises minimally invasive procedures to diagnose and/or treat a wide range of emergency and elective conditions. Interventional radiology can be an alternative to conventional surgery and has been shown to decrease morbidity, shorten hospital admissions and decrease medical expenses. In some instances, IR procedures are the only feasible treatment for patients unfit for conventional surgery.

There has been no evaluation of the impact of COVID-19 on SA IR services. Such an assessment would be beneficial. It would identify any potential clinical backlog resulting from the pandemic, facilitate departmental planning for any future similar scenarios and contribute to the global discourse on IR services during the pandemic, highlighting any unique features in the SA context. The latter is particularly important, given the SA quadruple burden of disease which includes communicable and non-communicable diseases, trauma, and maternal and child health. Additionally, given that IR is a relatively new SA subspecialist imaging discipline, it will enhance our understanding of the IR service generally.

### Aim

This study aimed to assess the impact of COVID-19 on IR procedures performed at a tertiary-level public sector SA hospital.

## Research methods and design

### Study design

A review of TBH IR procedures performed on adults (18 years and older) over three 9-week periods in 2020: period (1) pre-strict lockdown (23 January–25 March), period (2) strict lockdown (26 March–27 May) and period (3) post-strict lockdown (28 May–30 June), which correlates with the government-defined lockdown periods.

### Study setting

The setting of the study is TBH IR suite.

### Study population and sampling strategy

The data included all adult (18 years and older) TBH IR procedures performed by radiologists during the specified periods. All IR procedures performed outside the study period, on children, or by other medical disciplines were excluded.

### Data collection and analysis

A customised search of the TBH integrated picture archiving and communication system-radiology information system (PACS-RIS) was conducted for relevant data on all IR procedures in the review period. Captured data were stratified by patient demography and by procedure description (vascular or non-vascular), indication (trauma or non-trauma), timing (normal or after hours) and location (IR suite or ward). Vascular procedures were further classified as diagnostic alone, or with an intervention such as angioplasty, stenting or embolisation. Non-vascular procedures included biliary or urinary decompression, abscess drainage and biopsies. Trauma procedures included any blunt or penetrating injury as the cause for presentation; otherwise, procedures were classified as non-trauma presentation. The period 08:00–16:00 Monday through Friday constituted ‘normal’ working hours. Any other time, including public holidays, was ‘after-hours’. Procedures performed outside the IR suite were regarded as bedside. Where PACS-RIS clinical information was incomplete, this was supplemented by details from the comprehensive TBH electronic clinical database.

### Statistical analysis

Descriptive statistics such as frequencies and percentages were compiled for each period. The chi-square test was used to test for differences in the distribution (proportions) between periods. A negative binomial regression model was used for comparison of the total number of procedures between the three 9-week periods, adjusting for age, gender and clinical indication, utilising a significance level of 5%.

### Ethical considerations

The study was approved by the Stellenbosch University Health Research Ethics Committee (S23/01/018). Patient data were anonymised, and confidentiality was ensured using a unique study identifier known only to the principal investigator.

## Results

**FIGURE 1:**
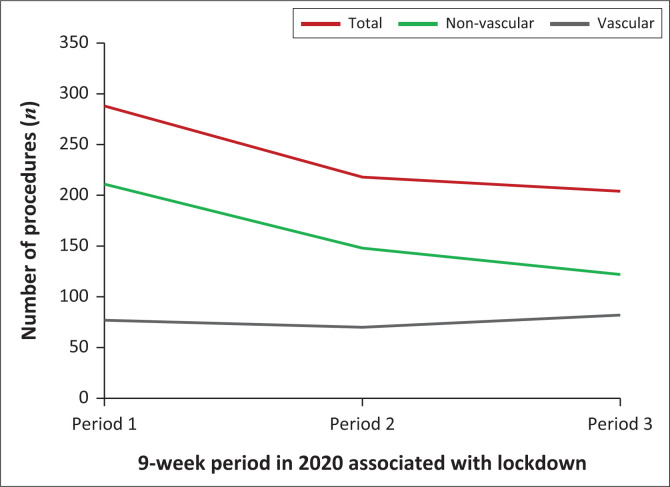
Number of procedures per period.

There were 288, 218 and 204 procedures performed in periods 1, 2, and 3, respectively (see Figure 1 and Table 3).

**TABLE 3:** Procedures and indications for procedures per period.

Procedures	Variable	Period 1	Period 2	Period 3	% difference 1 vs 2	% difference 2 vs 3	% difference 1 vs 3	Incidence rate ratio period 1 vs 2	Incidence rate ratio period 2 vs 3	Incidence rate ratio period 1 vs 3	*p*-value		
											Cl period 1 vs 2	Cl period 2 vs 3	Cl period 1 vs 3
**Overall**													
Total	*n*	288	218	2004	−24.3	−6.4	−29.1	-	-	-	-	-	-
	%	100	100	100	-	-	-	-	-	-	-	-	-
	Mean/day	4.6	3.5	3.2	-	-	-	0.757	1.069	0.708	0.002	0.496	0.001
Normal hours	*n*	252	192	182	−23.8	−5.2	−27.8	-	-	-	-	-	-
	%	87	88	90	-	-	-	-	-	-	-	-	-
	mean/day	4.0	3.0	2.9	-	-	-	0.762	1.055	0.722	0.004	0.606	0.001
Interventional suite	*n*	285	215	202	−24.5	−6.0	−29.1	-	-	-	-	-	-
	%	99	99	99	-	-	-	-	-	-	-	-	-
	mean/day	4.5	3.4	3.2	-	-	-	0.754	1.065	0.709	0.002	0.525	0.001
Males	*n*	160	127	117	−20.6	−7.9	−26.9	-	-	-	-	-	-
	%	55	58	57	-	-	-	-	-	-	-	-	-
	mean/day	2.5	2.0	1.9	-	-	-	0.794	1.085	0.731	0.052	0.523	0.010
Median age (years)	*n*	49.8	47.9	49.9	−4.0	4.4	0.2	-	-	-	-	-	-
**Non-vascular**													
Total	*n*	211	148	122	−29.8	−17.6	−42.2	-	-	-	-	-	-
	%	73	68	60	-	-	-	-	-	-	-	-	-
	mean/day	3.3	2.3	1.9	-	-	-	1.426	0.701	0.578	0.001	0.590	< 0.001
Collection drainage	*n*	97	67	54	−30.9	−19.4	−44.0	-	-	-	-	-	-
	%	34	31	26	-	-	-	-	-	-	-	-	-
Urinary/biliary drainage	*n*	85	59	60	−30.6	1.7	−29.4	-	-	-	-	-	-
	%	29	27	29	-	-	-	-	-	-	-	-	-
Biopsy	*n*	29	22	8	−24.1	−63.6	−72.4	-	-	-	-	-	-
	%	10	10	4	-	-	-	-	-	-	-	-	-
**Vascular**													
Total	*n*	77	70	82	−9.1	17.1	6.5	-	-	-	-	-	-
	%	27	32	40	-	-	-	-	-	-	-	-	-
	mean/day	1.2	1.1	1.3	-	-	-	defer	defer	defer	0.999	0.410	> 0.999
Diagnostic alone	*n*	36	36	43	0	19.4	19.4	-	-	-	-	-	-
	%	13	17	21	-	-		-	-	-	-	-	-
Diagnostic with intervention	*n*	41	34	39	−17.1	14.7	−4.9	-	-	-	-	-	-
	%	14	16	19	-	-	-	-	-	-	-	-	-
**Indications for procedures**													
Non-trauma	*n*	263	189	174	−28.9	−7.9	−33.8	-	-	-	-	-	-
	%	92	87	85	-	-	-	-	-	-	-	-	-
	mean/day	4.2	3.0	2.8	-	-	-	defer	defer	defer	0.410	0.013	0.496
Trauma	*n*	25	29	30	16.0	3.4	20.0	-	-	-	-	-	-
	%	8	13	15	-	-	-	-	-	-	-	-	-
	mean/day	0.4	0.5	0.5	-	-	-	1.16	1.2	0.967	0.590	0.987	0.504

vs, versus; Cl, confidence interval.

During period 2, the overall, non-vascular and vascular procedures decreased by 24% (*p* = 0.002), 30% (*p* = 0.001) and 9% (*p* = 0.999), respectively (see Table 3). During period 3, the overall and non-vascular procedures declined by a further 6% (*p* = 0.496) and 18% (*p* = 0.001), respectively, while vascular procedures increased by 17% (*p* = 0.410) (see Table 3). In period 3, the overall and non-vascular procedure totals were 29% (*p* = 0.001) and 42% (*p* ≤ 0.001) lower than period 1 levels, whereas vascular procedures exceeded period 1 by 7% (*p* ≥ 0.999), the latter thereby demonstrating the so-called ‘rebound phenomenon’ (see Table 3). This ‘rebound phenomenon’ is defined by the initial decrease in procedures from period 1 to period 2. This is subsequently followed by an increase in procedures from period 2 to period 3, so much so that the number of procedures in period 3 is greater than the number of procedures in period 1 (see Figure 1).

In period 1, non-vascular procedures constituted almost three-quarters (*n* = 211,73%) of all procedures, compared to 68% and 60% in periods 2 and 3, respectively (see Table 3).

Collection drainages progressively declined by 31% and 19% in periods 2 and 3, respectively. While biliary and urinary drainages decreased by 31% during period 2, there was no further decline in period 3. Biopsies, broadly representing the diagnostic oncology service, were the most impacted TBH IR component, showing a progressive decline of 24% and 64% in periods 2 and 3, respectively (see Table 3).

The diagnostic vascular studies alone showed no decline during period 2. In period 3, diagnostic alone and those with intervention increased by 19% and 15%, respectively (see Table 3).

Non-trauma procedures predominated in all periods, declining by 29% (*p* = 0.410) and significantly 8% (*p* = 0.013) in periods 2 and 3, respectively, with period 3 lower than period 1 (34%; *p* = 0.496). A striking feature was the progressive increase in the proportion of trauma-related procedures across the three periods, with an increase in periods 2 and 3 of 16% (*p* = 0.590) and 3.4% (*p* = 0.987), respectively (see Table 3).

## Discussion

This study provides the first detailed analysis of the impact of COVID-19 on public sector IR services at the institutional level in sub-Saharan Africa (SSA). As such, it addresses an important knowledge gap. This was highlighted in the 2021 systematic review of the impact of the COVID-19 pandemic on IR services worldwide, which included no SSA data, while underscoring the importance of institutional analyses from different settings, geographic locations, and regulatory frameworks globally.^16^

In common with other comparable analyses, in private and public health systems, we recorded decreased overall procedures during the strict lockdown. The magnitude of our institutional decrease of 24% was in the lower range of decreases documented to date, being comparable to the 31% recorded across six United Kingdom (UK) institutions and the 32% documented in a large United States healthcare system.^22,23^ However, it was lower than the 42% and 48% documented in Egypt and Italy, respectively, and substantially lower than the 82% recorded in China.^17,24,25^ The differential impact of COVID-19 across healthcare systems and institutions may reflect variations in the characteristics of the pre-COVID-19 caseload. It is well recognised that elective and outpatient IR procedures were most impacted globally.^16^ Given the resource constraints of the SA public healthcare sector, the TBH IR service has historically been confined to emergency and urgent cases, possibly accounting for the lower impact than on centres with a greater proportion of elective cases pre-COVID-19. This is of value when considering the urgency of procedures performed during a pandemic in respective IR centres. In an elective procedure-based IR centre, personnel can be de-escalated or redeployed, whereas in an emergency procedural IR, personnel need to be maintained, although fewer procedures are expected.

A feature of the COVID-19 pandemic across the broad range of medical disciplines, globally, has been the tendency for patient volumes in the immediate post-lockdown period to be lower than the pre-COVID-19 period.^18,19,26,27^ This trend has certainly been documented for IR procedures.^25^ Our finding of a further decrease in the overall IR caseload on the relaxation of the strict lockdown is thus in keeping with international trends. It reflects Western Cape Government and TBH policies whereby outpatient clinics and operating theatres were not running at full capacity even after the relaxation of the strict lockdown. It also likely reflects a change in patient behaviour regarding healthcare seeking that has been documented globally post COVID-19. This demonstrates that patients delay presenting to healthcare facilities. The reasons for this are multi-factorial, but include financial hardship, decreased access to public transport and fear of COVID-19 exposure.^23^ This also highlights the need for government policy regarding more efficient communication to the population regarding the need for presentation to healthcare facilities during a relaxation of the strict lockdown.

In attempting to understand trends identified in the various sub-analyses of our study, the progressive decline in *collection drainages* is intuitive, given that drainable collections typically relate to prior surgery, and surgical lists were curtailed both during and after the strict lockdown. *Biopsies*, broadly representing the diagnostic oncology service, were the most impacted TBH IR component, showing a progressive decline which was particularly marked during the post-strict lockdown. This likely reflects the cumulative effect of decreased access to outpatient facilities, limitations to public transport, changes in healthcare-seeking behaviour and the national policy to suspend oncology screening services even after the strict lockdown.^5,28^ Our institutional decrease in *biopsies* across all periods by 72% is higher than the 36% decrease recorded for six common cancer diagnoses in the public sector pathology laboratories of the Western Cape province between April 2019–June 2019 and April 2020–June 2020.^29^ Our institutional figures are also higher than those of Belgian pathology laboratories, which recorded a 44% reduction in total diagnoses of invasive cancers between April 2019 and April 2020, but are slightly lower than the US COVID and Cancer Research Network study, which recorded a 74% decrease in new cancers diagnosed between April 2019 and April 2020.^27,30^ It is noteworthy that biopsies were proportionally the most affected component of our IR service, whereas the systematic review of IR services across the globe showed that most centres preserved interventional oncology capacity through the pandemic.^15,28^ This demonstrates a decrease in biopsy and collection drainage procedures that could have an impact on training junior colleagues in obtaining these procedural skills.

Our finding that *biliary and urinary drainages* decreased during period 2 but showed no further decline in period 3 likely highlights the minimally invasive and innovative nature of IR, with the potential to serve as a viable alternative to traditional and laparoscopic surgery in the face of curtailed surgical lists. This role was underscored by Zhong and coworkers in the UK, who recorded a 66% increase in cholecystostomies during the pandemic, following decreased access to laparoscopic surgery.^22^ Regarding *urinary drainage procedures*, this study demonstrated a slightly higher reduction in urological procedures of 30% when compared to the US percentage of 5% – 25%.^31^ In the US, the decline in urological procedures was the result of an interruption in outpatient visits.^31^ This analogy correlates with the decline in our institute’s IR urological service relating to the closure of outpatient clinics and cessation of elective surgical lists.

Although there was an *overall* modest (9%) decrease in vascular studies during the strict lockdown, *diagnostic studies alone* showed no decline, likely reflecting the compelling clinical setting and emergent nature of critical limb-threatening ischaemia, warranting immediate work-up. The 17% increase in vascular studies during the post-strict lockdown, constituting the so-called ‘rebound’ phenomenon, likely mirrors the rebound in trauma-related emergency unit admissions in the Western Cape province in the post-strict lockdown period. One Western Cape public sector trauma unit recorded 115% and 124% increases in gunshot and stab wound admissions, respectively, during the post-strict lockdown.^10^ This highlights the need for possible policy adaptation in an IR during a pandemic, so that more vascular-trained personnel and dedicated theatre time are made available during these periods to manage the increase in vascular procedures.

This study showed a 16% and 3.4% increase in trauma-related IR procedures during and post the strict lockdown, respectively. In the Western Cape during lockdown periods, a rebound phenomenon in emergency trauma admissions was described.^10^ This does not correlate with our institute’s IR services, which managed a constant increase in trauma procedures. This may relate to variable recruitment of emergency unit and trauma surgery personnel to COVID-19 wards during different lockdown periods, who would have previously managed these procedures. As a result of the lack of staffing in these areas, there was a ‘spillover’ effect, with trauma procedures therefore managed by the IR unit. This underlines the importance of a functional emergency IR unit during a pandemic, especially given the change in staff allocation during lockdown periods. This demonstrates a procedural shift from the surgical department to the IR department.

### Strengths and weaknesses

Limitations included the use of data from a single IR department in a single health system, thereby limiting the depth of analysis, that is, a lack of multicentre data. However, the study was based in the largest public sector tertiary hospital in the Western Cape province, allowing for a large study sample and the full spectrum of IR procedures performed in our resource-limited setting. This study did not focus on the outcome-based measures of the procedures (i.e. morbidity and delayed diagnosis) as this is not the primary responsibility of the emergency IR in our given centre. Taking this into account, this is a limitation of the study as it does not define the impact of the procedures performed. This study was performed retrospectively, which could have decreased the comprehensiveness and accuracy of data collection. However, data were systematically extracted from the robust institutional PACS-RIS database. This study did not compare annual or seasonal variations across the three periods, thereby adding the possibility of confounding factors of seasonal variation as well as pandemic variation measures. The impact of cyclical annual or seasonal variations in morbidity was thus not incorporated in the analysis.

A strength is that it represents the only work to date evaluating the impact of COVID-19 on IR services in SSA. This study contributes to the global discourse on IR services during the pandemic and highlights unique features in the SA context.

## Conclusion

This study provides unique insights into the impact of COVID-19 on IR services in SSA. It also affords a broader perspective on the emerging role of IR in health systems across the continent.
